# A Conceptual Model Map on Health and Nutrition Behavior (CMM^HB/NB^)

**DOI:** 10.3390/ijerph17217829

**Published:** 2020-10-26

**Authors:** Kirsten Schlüter, Sandra Vamos, Corinne Wacker, Virginia D. E. Welter

**Affiliations:** 1 Institute of Biology Education, University of Cologne, Herbert-Lewin-Str. 2, 50931 Cologne, Germany; cwacker1@uni-koeln.de (C.W.); virginia.welter@uni-koeln.de (V.D.E.W.); 2 School of Interdisciplinary Health Programs, Western Michigan University, 1903 W Michigan Ave, Kalamazoo, MI 49008-5382, USA; sandra.vamos@wmich.edu

**Keywords:** health literacy, food literacy, behavior change theories, knowledge–attitude–practice, KAP, Maslow’s pyramid of needs, intention–behavior gap, attitude–behavior gap, concept map

## Abstract

The Conceptual Model Map (CMM) presented here is intended to show the connections between different theories and constructs in the field of health and nutrition behavior (including literacy models, the knowledge–attitude(s)–practice(s) survey structure (KAP), behavior change theories, and Maslow’s pyramid of needs). The CMM can be used as a map to locate existing and future studies, to show their range of effect as well as their limitations. In this context, the CMM also reveals reasons for the attitude/intention–behavior gap.

## 1. Introduction

In the health sector, there are many different theories, concepts, and models as well as associated studies. They all serve to explain individuals’ health and nutrition behavior and to develop appropriate interventions on a theoretical as well as empirical basis. These different approaches each certainly have their own justification. However, they also have certain focal points and therefore cannot always reflect the entire reality. On the other hand, various health behavior theories address similar influencing factors, but use different terms [1]. We aim to illustrate this diversity of theories, show their different focal points and distinctions from one another, but also their areas of overlap. By this, the complexity of human behavior as well as associated difficulties of behavior change should be visualized.

**Figure 1:**
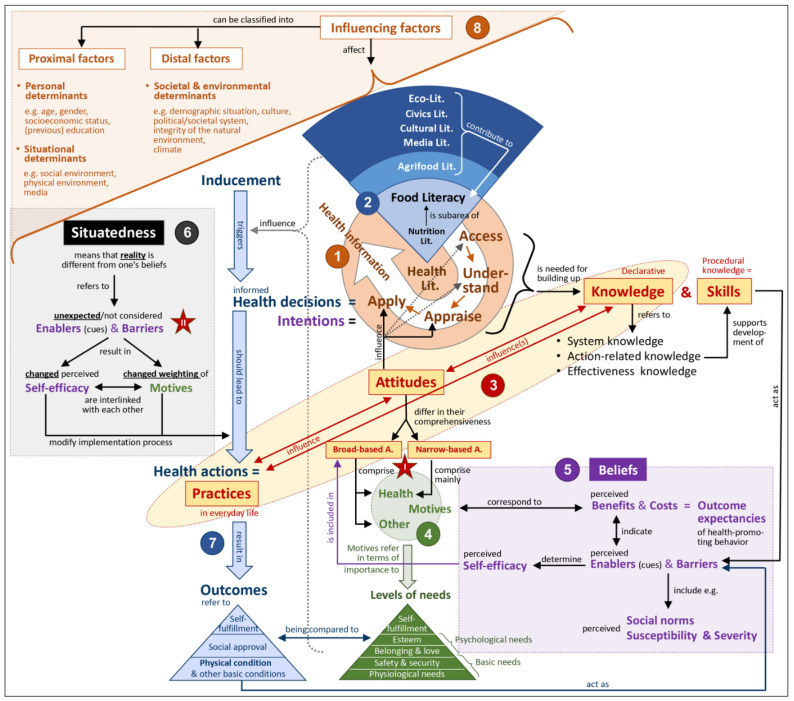
Conceptual Model Map of factors influencing Health and Nutrition Behavior (CMM^HB/NB^). The circled numbers serve as reading hints and refer to the headings in the text. The two red stars refer to possible explanations for the attitude/intention–behavior gap (see Section 2.4, Section 2.5 and Section 2.6 in the text).

The product of our theoretical work is a **C**onceptual **M**odel **M**ap on factors influencing **H**ealth and **N**utrition **B**ehavior (CMM^HB/NB^). The term CMM is an acronym being composed of the following three components: (1) CM_1_ = Conceptual Models (in the form of theories and constructs): They form the content basis of the CMM; (2) M_1_M_2_ = Model Map: By bringing different conceptual models together, a visual map was created on which the range of model content and the areas of overlap are shown; and (3) CM_2_ = Concept Map: This is a didactic method by which the knowledge structure of a specific content area is presented in a graphic way [2]. It is composed of specific structural elements. Important terms of the content area (so-called nodes) are connected by aligned lines (arrows), which are labeled to indicate the relation between the terms [2]. The CMM on health and nutrition behavior presented here is designed in the format of a concept map (see Figure 1).

The development of the CMM can be seen as a qualitative process. In order to bring in different areas of expertise, we worked together in an interdisciplinary group. It consisted of one person each from the following areas: education, public health, biology, and psychology. In a first step, we compiled selected theories, models, and even survey strategies from different research areas: public health, psychology, and education. The selected theories and models are well-established in the scientific community. Thus, they can be found in basic textbooks and encyclopedias (behavioral change theories [3,4,5], pyramid of needs [6,7]), or they are a product of systematic literature reviews (literacy concepts [8,9]), or present a common survey practice (KAP [10]). We analyzed the components of the selected theories and models regarding their definition. Based on these definitions, we discussed and determined areas of overlap in order to link the theories and models. For this merging process, supplementary studies had to be integrated (e.g., from motivation research) [11], some definitions had to be adapted, and additional explanations were necessary to create a coherent overall picture of influencing factors on health behavior. The additional explanations we added (e.g., distinction between two types of attitudes) were based on personal experience only. Therefore, these explanations can be regarded as hypotheses that have yet to be empirically tested. The selection of the theories and models and their recombination to an overall explanation for health behavior was an iterative and interactive process in which suggestions made by one person were critically reflected and commented on by the co-authors. Then, the resulting extensions and changes in the CMM again went through a process of discussion and adjustment until the authors reached agreement.

The necessity of developing the CMM can be justified in three ways. (1) Communication: When working together in an interdisciplinary team, there may be comprehension difficulties caused by the different research backgrounds of the individuals. Therefore, it is necessary to come to a common understanding of terminology, intended goals, and appropriate study design. The CMM brings together findings from different research areas (public health, psychology, and education) and thus creates a common framework of understanding. (2) Research: Existing health research projects can be located within the CMM, and the possible scope of their explanatory and predictive power concerning health and nutrition behavior can be roughly estimated. Likewise, the CMM can be used for planning purposes to determine which variables to examine in order to find out whether intervention measures will be effective. (3) Intervention: The CMM can be used to develop new interventions. It points out different possible starting points but also potential challenges in advance regarding the effectiveness of interventions.

## 2. The Conceptual Model Map

Figure 1 shows the Conceptual Model Map on Health and Nutrition Behavior (CMM^HB/NB^) we developed, which is explained below. Due to the complexity of the CMM, its various parts are numbered and colored differently. Key terms that either appear in the CMM or are of explanatory or structuring importance are printed in bold in the following model description.

### 2.1. The Orange Circle (No. 1)/Health Literacy

The core of the CMM refers to the Integrated Model of **Health Literacy (HL)** of Sørensen et al. [8]. This model was chosen because it is based on a systematic literature review on HL. Furthermore, this model visualizes four central processes/skills on the individual level that appear in a similar way in many definitions of HL [12]. These processes/skills refer to gaining access to health information, understanding it, critically evaluating/appraising it, and applying it to make informed decisions. The mentioned processes can be regarded as methodological elements (comparable to tools) that are necessary prerequisites for building up any literacy. The content-specific element is the health-related information to which these general processes (tools) refer. We understand health information to include both theoretical parts being mainly language- and paper-based as well as practical parts referring to hands-on demonstrations (e.g., performing yoga exercises, using kitchen utensils for preparing a meal). Thus, if a person is health literate, it means that he or she has developed content-specific (declarative) **knowledge** and (procedural) **skills**, which allow an individual to act in a health-promoting way. However, if an individual is in some way health-literate, this does not necessarily imply that the person is actually using his or her health-related knowledge and skills in everyday life. In our view, the motivation to do so does not arise automatically. Rather, it depends on the attitude of a person and the particular situation in which this person finds him or herself.

The four central processes for literacy development are expressed using brief statements whereby our descriptions slightly differ from those of Sørensen et al. [8]. **Access** refers to knowing how and where to find theoretical and practical information on the relevant subject matter. **Understand** refers to the ability of making meaning from the information obtained. While this term is appropriate for processing theoretical information, it is less appropriate for practical information. In the latter case, one should rather speak of imitating as accurately as possible what had been demonstrated. **Appraise** refers on the one hand to being able to assess the quality of the available information (whether it is credible and up-to-date), and on the other hand to judge whether the information is relevant and applicable for oneself. We also believe that the term “**apply**” can have different facets of meaning. For example, on the one hand, the aim is to practice implementation of the gathered (health) information, whereby skills will be developed. Such training tasks can be seen in school health and patient education (see e.g., standard 7 of the Joint Committee on National Health Education Standards [13]). On the other hand, applying can be interpreted as making an informed decision whether and to what extent (the gathered, assessed, and tested) health information shall be incorporated in one’s everyday life [8]. In both cases, the term apply denotes an intermediate stage on the way to behavior change. Since a person’s actual behavior may differ from his or her literacy level (which is one’s knowledge and skills), we do not consider it appropriate to equate the term apply with everyday behavior. With reference to the second meaning (making an informed decision), we would rather equate the term apply with the formation of an **intention** to act (or not to act).

### 2.2. The Blue Circle Segment (No. 2)/Food Literacy

This part of the CMM refers to the **Food Literacy (FL)** Conceptual Model of Hernandez et al. [14,15]. Even if there are other models, e.g., [16,17,18], and this one is just in the stage for a publication, we refer to it for different reasons. (1) This latest FL model is based on existing reviews, including systemic and scoping ones, and it considers their results in the modeling process. (2) There is a clear reference to HL because the core element of this FL model is similar to that of Sørensen et al. [8]. That means that it refers to the four central processes of accessing, understanding, appraising, and applying health-related nutrition information through which literacy is acquired. (3) In addition, the FL model is based on an interdisciplinary approach, which is why it includes various other literacy areas referred to below.

The close connection between HL and FL is given by the field of **nutrition literacy (NL)**. NL corresponds to HL, but it is restricted to a specific health content, namely nutrition [9,19]. While NL mainly refers to basic nutrition information (e.g., national nutrition guidelines), FL goes beyond that. Thus, NL is considered a sub-area of and prerequisite for FL [9,20]. In other words, FL is an extension of NL in several ways: FL also includes food preparation skills, the shopping and storage of food, as well as judging food quality [9,20,21]. In addition, FL comprises an understanding of the potential cultural, political, social, economic, and environmental impacts of food decisions [9,22,23]. Thus, FL is influenced by diverse areas of other literacies [14,15]—whereby this of course also applies to HL. For example, **agri-food** (or agricultural) **literacy** may affect one’s choice of food. It comprises information about food supply matters, such as the production (animal farming and plant cultivation), processing, marketing, and distribution of agricultural products, the need of natural resources for these purposes, etc., [24,25]. Moreover, media are an important source of information about food [17]. However, this information has to be critically analyzed, e.g., with regard to its origin, intention of publication, and trustworthiness—which are skills belonging to **media literacy** [26,27]). **Cultural literacy** refers to knowing that society as well as oneself are shaped by “collective beliefs, customs, world-view, and social identity” [28] (p. 197)—and this is also true for our eating habits. **Civic literacy** means that citizens are trained in their ability to perceive problems in public life—which also includes the food sector—as well as in participating in the decision-making processes for problem solving [28]. One way of exerting social influence can be done through NGOs, for example, such as consumer protection centers. Finally, there is the area of ecological or **eco-literacy**, which involves understanding the mutual relationships and interdependencies of people among each other and with their environment and how to make this relationship sustainable [29,30]. By taking all these additional literacy areas together into account, the development of a **critical** food/health **literacy** may be promoted [9,31,32].

### 2.3. The Yellow Ellipse with Red Writing (No. 3)/KAP

Another central element of the CMM is the **KAP** survey structure with its three components: knowledge (K), attitudes (A), and practices (P) [10]. The **knowledge** component can be divided into two main types: **declarative** and **procedural** knowledge. This subdivision is based on the distinction between two types of long-term memory: declarative memory, which refers to facts and events, and non-declarative memory, which includes as a subunit procedural memory, referring to skills and habits [33]. Very abridged, there is a distinction between **factual knowledge** and **skills**, whereby the type of knowledge acquired at least partly depends on whether information is conveyed theoretically or practically. Derived from the environmental field and transferred to the health area, factual knowledge can be further differentiated into three subtypes [34,35]: **systemic knowledge** relates to a system’s structure, function, adaptability, and vulnerability, with the human body representing such a system. **Action-related knowledge** refers to what we can do to keep or get our body healthy. This kind of knowledge, even when conveyed in a descriptive manner, may assist the development of practical skills provided that the theoretical information is put into practice. **Effectiveness knowledge** finally indicates which course of action is particularly promising in order to achieve a health goal. All types of knowledge can be built up by the four central processes that form the basis for developing HL/FL, namely by accessing, understanding, appraising, and applying health-related nutrition information.

The second component in the KAP survey structure, the **attitudes**, are defined as “evaluative feelings, favorable or unfavorable, toward particular objects” [36] (p. 32). When associating the health literacy model [8] with the KAP survey structure, it is the attitude component of KAP that affects the four central processes leading to HL/FL by influencing the motivation to perform these processes. The effect of one’s attitude may be even stronger on the two later processes, which are appraising and applying. We believe that appraising a behavior recommendation is a reflection of a person’s attitude, and the same applies to the decision of whether to integrate the proposed behavior into one’s everyday life (= applying health information), which is a direct consequence of the preceding appraisal. The third component of the KAP survey structure refers to **practices** that are the actual behavior a person shows in everyday life.

The three components of the KAP survey structure are assumed to influence each other mutually [36]: Knowledge is thought to influence attitudes more than behavior/practices, because changing behavioral patterns involves more effort. Attitudes can have an effect on behavior but can also affect the extent or depth of the knowledge acquired. A changed behavior has an effect on the attitude by changing it in such a way that it supports the new behavior [36]. Likewise, a changed behavior can expand the action-related knowledge through new practical experiences that have been gained.

### 2.4. The Green Components (No. 4)/Decision Motives and Pyramid of Needs

In the CMM, we assume **attitudes** to correspond to the sum of a variety of positively as well as negatively evaluated decision criteria, which are referred to as **motives**. Hereby, each motive has its own magnitude of relevance. Thus, motives have an orientation (positive or negative in relation to a given goal, such as implementing a healthy diet) and a weighting factor. Depending on the number and thematic range of these motives on which an attitude is grounded, we distinguish between broad- and narrow-based attitudes. **Narrow-based attitudes** refer to relatively few motives that are closely related to the given objective—in this case, a health-promoting diet. Thus, the health aspect or health motive is very likely to be at the forefront when someone expresses his or her attitude. This is different for **broad-based attitudes**, which result from a large number of decision criteria taking not only the goal of maintaining good health into account, but also addressing several other target dimensions.

With reference to these two types of attitudes, a possible explanation for the well-known **attitude–behavior gap** [37] can be deduced (see red star I in Figure 1). While a narrow-based attitude toward health actions considers primarily the importance of health motives, it disregards the other motives that guide action. Therefore, a narrow-based attitude often shows little conformity with an individual’s behavior.

In a cross-national study on reasons for dietary choice [11], eleven other motives next to health were analyzed in terms of their importance, namely liking, need and hunger, habits, convenience, pleasure, natural concerns, traditional eating, sociability, price, weight control, and visual appeal (of foods), with the first three being given even greater importance than the health motive [11]. However, even this list of motives is not complete. In the various Health Behavior Theories [1], additional variables influencing a person’s health-promoting behavior are included (e.g., perceived social norm, perceived thread = susceptibility and severity of getting ill), which should be integrated into the previous listing of motives. In this way, a broad-based attitude may be determined.

We assume that the relevance of the various motives at least partially to correspond to **Maslow’s pyramid of needs** [38]. Thus, pursuing health goals will only be of increased relevance when a person’s health is no longer given and there is a deficit at the basic levels of physiological and safety needs (levels 1 and 2 of Maslow’s pyramid). On the other hand, if a person is in a good state of health and healthy eating is only a preventive measure, then a healthy diet may only fulfill an advanced level of need, such as that of self-fulfillment/self-actualization (level 5).

### 2.5. The Lilac Rectangle (No. 5)/Beliefs in Behavior Change Theories

This part of the CMM refers to different types of **beliefs** mentioned in different Health Behavior Theories, such as the Health Belief Model (HBM) [39,40], the Theory of Planned Behavior (TPB) [41], and the Social Cognitive Theory (SCT) [42]. Despite differences in terminology, the beliefs mentioned in these theories are quite similar [1]. All these different beliefs (such as perceived benefits and barriers, social norms, self-efficacy, and susceptibility and severity to food-related illnesses) can serve as justifications for a person’s attitude. The aforementioned decision motives are closely related to these beliefs, e.g., to the assumed **benefits** and **costs**—and thus to the **outcome expectancies**—concerning health-promoting (nutrition) behavior. For example, if a person considers price to be an important decision criterion/motive in food selection, then healthy foods offer an additional benefit (besides the obvious one of being healthy) if they are considered cheap. On the other hand, healthy foods are at a disadvantage if they are considered expensive. Thus, the price can become an **enabler** or a **barrier** to buying healthy food. In general terms, the assumed (motive-specific) benefits and costs of a healthy (eating) behavior can point to corresponding (motive-specific) enablers or barriers of this behavior. It is important to note that this chain of argumentation can also be reversed when starting with possible enablers and barriers of health-promoting behavior, such as family support or peer rejection (being based on **social norms**). These may affect the perceived benefits and costs (e.g., belonging to a social group or exclusion from this group), which in turn may influence the priority of a person’s decision motives regarding whether or not to adopt the considered health-promoting behavior. Assumptions about one’s own **susceptibility** to and the perceived **severity** of diet-related diseases can also act as enablers or barriers. These assumptions become a barrier whenever a person overestimates his or her resistance to a behavior injurious to health. Further barriers are listed in a study of Truman and Elliott [43] including a lack of skills and abilities to carry out the desired nutritional behavior, a lack of resources (time, money, equipment), and hindering conditions of the social environment whereby the latter can be understood as being equivalent to social norms.

If one offsets all the various enablers and barriers of health-promoting behavior in their different magnitudes, one will obtain—in our opinion—a measure of **self-efficacy**. However, what is the connection between self-efficacy and attitudes? We assume self-efficacy to contribute especially to a broad-based attitude, since the latter includes all possible decision motives and thus ultimately also the enablers and barriers of a health-promoting behavior, which form the basis of one’s self-efficacy. In contrast, self-efficacy is assumed to be barely included in a narrow-based attitude, because the latter primarily considers the importance of the health motive but it hardly considers other motives referring to potential barriers of health-promoting behavior (see the red star I in Figure 1). Therefore, a positive narrow-based attitude (“A healthy diet is important for me.”) may be opposed to a low self-efficacy (“I think I will not be able to implement a healthy diet.”) and result in a non-performance of health-promoting behavior. In this case, there is an **attitude–behavior gap**. This gap is particularly likely to occur when a person with low self-efficacy expresses a narrow-based attitude. Whether a person, when asked about his or her attitude toward a particular behavior, expresses a broad- or narrow-based attitude will probably depend on personal characteristics, such as the degree of critical reflection on the action to be considered.

### 2.6. The Grey Rectangle (No. 6)/Situatedness of Actions

The apply step in the health literacy concept of Sørensen et al. [8] can be interpreted as making an informed decision. Thus, it is equivalent to forming of an **intention** to act. However, such an intention does not always correspond to a person’s actions. An important reason for the **intention–behavior gap** (see the red star II in Figure 1) is that reality sometimes looks different from what one would have thought in theory [37,44,45]. Unforeseen and unconsidered barriers can arise, but there can also be unexpected supportive offers (enablers). These unforeseen barriers and enablers might change the importance and balance of a persons’ (decision) motives as well as his or her self-efficacy whether he or she is able to implement the intended behavior. For example, the intention to cook and eat a light vegetable dish in the evening can be prevented by tiredness after a long working day, or the lack of time for healthy grocery shopping. Thus, the motives of recovery/sufficient sleep (level 1 of Maslow’s pyramid = physiological needs) or being afraid of disapproval from one’s employer if one leaves work too early or in between (level 2 of Maslow’s pyramid = security to keep one’s job) are more important at this very moment than preparing a “healthy” dinner (level 5 of Maslow’s pyramid = self-fulfillment/self-actualization, because the intended health measure has just a preventive effect and is not essential). Due to the unforeseen barriers, the self-efficacy of putting the stated intention (cooking a “healthy” dinner) into practice will decrease. However, there might be an unforeseen enabler, which could be a friend who spontaneously offers to do the grocery shopping and to cook together with you. The motive of having a “healthy” dinner is now supported by the motive of having a social event (level 3 of Maslow’s pyramid = love and belonging). With the support of the friend, the self-efficacy of cooking the intended “healthy” dinner at least on this one day also increases. With reference to this example, we introduce the term of **situatedness** [46], which is well known in the educational context. In our context, it means that any action is shaped or influenced by the special and changing conditions found in diverse everyday situations, with the conditions being either supportive or hindering. Another problem when transferring an intention into an action is that eating acts occur several times a day, and therefore, it is easy to postpone the intended “healthy” eating behavior over and over again. Generally speaking, the reason for the intention–behavior gap refers to a person’s inability of considering reality with its variety and complexity of situations (resulting in a changing weighting of decision motives) during one’s theoretical decision-making process. Therefore, the intention–behavior gap is also a **theory–reality gap**. The fact that the assessment of the importance of decision motives is different under theoretical conditions than in a concrete everyday situation was recently demonstrated by Wahl et al. [47] for the nutrition sector and was classified as an area for further research.

### 2.7. The Line of Blue Arrows (No. 7)/Phases of an Action Episode

This part of the concept model map represents an episode of action. There is an **inducement** at the beginning that demands health-relevant decisions of a person. For example, reasons can be the worsening of one’s own state of health, illnesses of other family members, or health information conveyed at school or by the media. The process from the inducement to the **decision** is influenced by one’s state of knowledge and one’s broad-based attitudes (taking into account all the different decision motives a person has). The subsequent process from the decision to the actual **action** is driven by the motives that are dominant in the present situation (and these may be motives or needs other than the care of one’s own health). If health promoting (or even detrimental) actions are carried out, they will lead to **outcomes** and have consequences. Bandura [42] distinguishes between three different outcome expectations: (1) physical; (2) social; and (3) self-evaluative, with the latter referring to self-satisfaction and self-worth. In our CMM, we use these categories of outcome expectations in order to categorize the actual outcomes. In this way, the results of health-promoting actions include not only the intended physical outcomes but also social consequences and a certain sense of self-fulfillment. These different outcome categories reveal similarity to the levels of **Maslow’s pyramid of needs**, if one would reduce the number of levels from five to three, namely basic, psychological, and self-fulfillment needs. In this way, a person can mentally make a comparison between his or her original needs and the outcomes obtained.

There is one aspect that is especially important in the evaluation of outcomes: The results of health-related actions go far beyond the health motive and also show consequences in other levels of needs and (decision) motives. Depending on the current situation, other needs and motives (e.g., pleasure, convenience, social acceptance among peers) may be more important than the health motive. This is especially the case when a person acts preventively, and therefore, the benefit of the behavior is not directly noticeable. Maintaining the current state of health is usually not considered a success, as no change is visible. Instead, health-promoting behavior may cause negative consequences for other, non-health-related needs and (decision) motives. These negative consequences as well as not noticeable health preservation effects may act as barriers and thus negatively influence one’s attitude toward continuing preventive behavior.

### 2.8. The Orange Triangle (No. 8)/Proximal and Distal Influencing Factors

A final part of the model map refers to various factors that influence a person’s health behavior (e.g., by acting as an inducement for a change in behavior, by influencing one’s level of knowledge or one’s attitude, or by acting as consciously perceived or unconsciously remaining enablers or barriers to health-promoting behavior). According to the models of Sørensen et al. [8] and Hernandez et al. [14,15], three groups of influencing factors are distinguished. **Personal determinants** refer to characteristic of a person such as gender, age, and genetic make-up, but also one’s education status, socio-economic status, and health status. **Situational determinants** describe a person’s social environment (e.g., family, colleagues, friends) as well as the physical one (e.g., the kind of food offered in a canteen at work) and corresponds at least partially to the perceived and actual barriers and enablers of one’s health behavior. Finally there are **societal and environmental determinants** which for example refer to the ethnic composition of a population, its traditions, the population density, the climatic conditions, the integrity of the natural environment, as well as state support services in prevention and health maintenance. All these influencing factors might affect a person’s level of nutrition literacy, attitude, and nutrition behavior.

## 3. Implications for Research and Practice

The Conceptual Model Map is intended to show the connections between different theories and constructs in the field of health and nutrition. It can be helpful for planning and interpreting research as well as for designing intervention measures, which will be explained in more detail below.

In terms of **research**, the CMM can help in two ways. (1) It can be used as a map to locate existing and future studies and to distinguish them from each other. Thus, the content range and the explanatory power of each study can be visualized. For example, a comparison of learners’ knowledge of different literacy areas (e.g., food literacy, agri-food literacy, and eco-literacy) may reveal whether there is a close linkage between them, meaning that high knowledge in one area may also lead to high knowledge in another field. However, as indicated by the map, such an investigation would not allow any statements to be made regarding health attitudes and actions. On the other hand, a KAP study would permit such statements to be made, since it surveys not only knowledge, but also attitudes and behavior. However, it is important to note that this type of study has its limitations, because the reasons for possible inconsistencies between the three areas (K, A, and P) remain unclear. Possible explanations can only be deduced if one knows the motives/beliefs on which an attitude is based and the specific conditions under which the behavior takes place, as illustrated in the CMM. (2) The CMM provides ideas for further research. Research questions may refer especially to those areas of the CMM where different theories and models are linked with each other by using explanations based on everyday experiences. Examples for future research questions are as follows: Can a distinction between narrow-based and broad-based attitudes be empirically proven? What does it depend on whether a person expresses a narrow- or broad-based attitude? Which (decision) motives do the interviewees themselves see as the basis for their attitude? How strongly does the weighting of these (decision) motives vary depending on the different action situations? To what extent, in relation to a specific situation, does the measured self-efficacy correspond to an offsetting of the perceived barriers and enablers?

The CMM can be used not only for research purposes but also for **intervention planning**. Two options are shown for this. (1) On the basis of the CMM, certain difficulties can be deduced regarding the effectiveness of interventions: 1(a) As has long been known, the transfer of knowledge and skills is a necessary but not sufficient measure to bring about behavioral change. 1(b) A negative attitude toward health measures can only be changed if one knows the (decision) motives and beliefs on which this attitude is based. 1(c) An expressed positive attitude toward health measures may not be as positive as it seems. This is because if it is a narrow-based attitude, then non-health-related (decision) motives are not taken into account in the formation of this attitude (see the red star I in Figure 1). 1(d) It is necessary to know the exact circumstances of different (everyday) situations in which the desired health-promoting behavior is to appear in order to think of specific measures for these situations that promote its occurrence. However, the problem is that the situations can change due to unforeseen events, and one might not be prepared for dealing with the changes (see red star II in Figure 1). 1(e) In order to increase the effectiveness of preventive and curative health measures, they must be adapted both to the characteristics of a person (including his or her motive basis—see above 1b/c) and to the particular circumstances of the action situation in which that person finds oneself (see 1d). Therefore, **individualization** (with regard to single persons) or **differentiation** (with regard to groups of persons) is necessary. (2) The CMM indicates different starting points for intervention. These refer—apart from classical information transfer—to strategies that should reduce the attitude/intention-behavior gap. None of the following proposals for intervention represent anything fundamentally new; however, using the CMM, it is possible to explain in a plausible way why these strategies may work. The explanations given are partly of a hypothetical nature and have to be tested by empirical studies. The first two intervention strategies (2a, 2b) refer to a potential **increase in self-efficacy**, while the following two (2c, 2d) refer to a **coupling of (decision) motives** that might result in a shift of one’s needs. The latter strategy (2e) finally focuses on an **exact action planning process**. The five strategies can be described as follows: 2(a) Reducing potential barriers of the desired behavior: This, for example, can be done by applying the small changes approach [48]. This approach proved that even small changes in eating habits and physical activity can reduce excessive weight gain and thus the risk of obesity [49]. Small behavioral changes have the advantage that they are easier to implement in one’s own lifestyle. 2(b) Promoting awareness and use of enablers for the desired behavior: This means that a person should consciously seek out people and situations that support him or her in implementing the desired behavior. A prerequisite for this pertains to one wanting to and being able to change the existing environment. 2(c) Increasing the costs for unfavorable behavior to be changed. For example, this could be done through the principle of self-commitment [50]. By telling others about one’s behavioral plans, an individual probably feels more bound to these plans, because otherwise (if one does not behave as publicly announced), a subjective view might evolve that others perceive one as unreliable or weak. By this, level 4 and perhaps even level 3 of Maslow’s pyramid of needs, i.e., being valued by others and belonging to others, may no longer be satisfied. Preventing the violation of these levels of need may in some cases be considered more important than adhering to habitual unhealthy behavior. The same mode of action may be found when rules are imposed on a person in the form of legal or institutional regulations (for example, smoking bans in public buildings and places as well as in restaurants). As soon as a person violates these rules, he or she must expect negative social (and pecuniary) consequences. 2(d) Providing additional benefits: If in everyday life, the motive of health prevention is of minor importance to a person, then it should be coupled with other motives that are of greater importance to this person. The more basic these other motives are according to Maslow’s pyramid of needs [38], the more effective a coupling should be—a hypothesis that requires investigation. This coupling of motives should increase the importance of the intended health measure, albeit for different reasons and not for the sake of health. For example, in a scenario, a person is advised to avoid heavily processed, high-calorie foods, such as fast food and convenience products. This person does not want to do so, as cooking by oneself prevents him or her from other leisure activities (e.g., meeting friends in the pub in the evening). A motive coupling may arise if the perceived tiresome cooking is combined with the satisfaction of other needs (than those of health prevention). Perhaps the music one listens to while cooking, as well as the delicious smell that arises during preparation, has a relaxing effect and distracts one from everyday stress (need for stress reduction). One might cook together with a friend in order not to be alone (need of belonging). One might receive recognition from colleagues and acquaintances because of the delicious meals one prepares (need of esteem). In our opinion, this area (coupling of motives) is an essential field of further research. It is important to find out which motive linkages are particularly attractive to learners, so that a requested health behavior does not stand on its own, but satisfies alternative needs at the same time. Thus, learners’ willingness to engage in preventive behavior might increase. 2(e) Action planning and coping planning according to Schwarzer [44]: An individual should plan the implementation of the desired, health-promoting behavior as precisely as possible by determining when, where and how it shall be realized. In addition, potential obstacles should be considered in advance as well as potential countermeasures. This precise planning is intended to ensure that when the situation simulated in one’s mind occurs, the requested health-promoting behavior is automatically retrieved and executed [51]. Such a planning process will at least partially include the previously mentioned implementation strategies (2a–2d).

Finally, it should be pointed out that the CMM presented here is not only applicable to health and nutritional behavior but applies in the same way to environmental behavior. All these areas show the problem of an attitude/intention behavior gap. This is because preventive health behavior, similar to protective environmental behavior, does not show any timely and obvious effects and thus hardly leads to a sense of achievement or satisfaction of personal needs. Instead, the positive effects develop gradually and over a long period of time.

## 4. Conclusions

The Conceptual Model Map (CMM) presented here uses established theories and models applied in the fields of public health, psychology, and education and integrates them into an overall model map. The linkages between these theories and models are partly based on assumptions that are still to be empirically tested and thus offer starting points for further research. Nevertheless, the CMM can already be used as a planning and reflection tool for health education measures and related research. Therefore, it is of interest to researchers, practitioners, and educators, but also policy makers. The relevance of the CMM extends beyond the content area described here in that it can also be applied to other content fields where there is an attitude/intention-behavior gap, such as environmental education.
